# Bacterial community structure in geothermal springs on the northern edge of Qinghai-Tibet plateau

**DOI:** 10.3389/fmicb.2022.994179

**Published:** 2023-01-31

**Authors:** Huai-sheng Zhang, Qing-da Feng, Ding-yue Zhang, Gui-lin Zhu, Li Yang

**Affiliations:** ^1^Center for Hydrogeology and Environmental Geology Survey, CGS, Baoding, China; ^2^State Key Laboratory of Biogeology and Environmental Geology, Wuhan, China; ^3^Tianjin Engineering Center of Geothermal Resources Exploration and Development, Tianjin, China; ^4^Institute of Agricultural Sciences in the Coastal District of Jiangsu Province, Yancheng, China

**Keywords:** geothermal springs, environmental response characteristics, Qinghai Tibet plateau, biogeochemistry, microorganisms in extreme environments

## Abstract

**Introduction:**

In order to reveal the composition of the subsurface hydrothermal bacterial community in the zones of magmatic tectonics and their response to heat storage environments.

**Methods:**

In this study, we performed hydrochemical analysis and regional sequencing of the 16S rRNA microbial V4-V5 region in 7 Pleistocene and Lower Neogene hot water samples from the Gonghe basin.

**Results:**

Two geothermal hot spring reservoirs in the study area were found to be alkaline reducing environments with a mean temperature of 24.83°C and 69.28°C, respectively, and the major type of hydrochemistry was SO_4_-Cl·Na. The composition and structure of microorganisms in both types of geologic thermal storage were primarily controlled by temperature, reducing environment intensity, and hydrogeochemical processes. Only 195 ASVs were shared across different temperature environments, and the dominant bacterial genera in recent samples from temperate hot springs were *Thermus* and *Hydrogenobacter*, with both genera being typical of thermophiles. The correlation analysis showed that the overall level of relative abundance of the subsurface hot spring relied on a high temperature and a slightly alkaline reducing environment. Nearly all of the top 4 species in the abundance level (53.99% of total abundance) were positively correlated with temperature and pH, whereas they were negatively correlated with ORP (oxidation–reduction potential), nitrate, and bromine ions.

**Discussion:**

In general, the composition of bacteria in the groundwater in the study area was sensitive to the response of the thermal storage environment and also showed a relationship with geochemical processes, such as gypsum dissolution, mineral oxidation, etc.

## 1. Introduction

The magmatic tectonic zone on the northern edge of the Qinghai-Tibet plateau has bred enormous and widely distributed thermal storage resources. More than kilometers of loose Pleistocene river–lake facies deposits were accumulated in the Gonghe Basin as a result of large-scale sedimentation in the early quaternary, which provided good spatial conditions for groundwater storage and resulted in the development of a convection-like geothermal system of medium-low temperature (Gao et al., 2018; Tang et al., 2020). The convection-type geothermal system at the medium-low temperature can in principle be considered as an open or semi-open geochemical system that performs various water-rock interactions. Much of the most recent research shows this system to also be an ideal location for the formation and enrichment of certain metals (including precious metal mineral resources, such as gold, platinum, rare earth, and even radioactive mineral resources) (Kozubal et al., 2008; Roychoudhury et al., 2013; Fortney et al., 2016).

The geothermal fluid is the primary carrier for the transmission of geothermal energy. They have a continuous and dynamic hydrologic relationship with the deep subsurface environment and carry a great deal of depth information (Deng et al., 2010; Cox et al., 2015; Zhang et al., 2019). Geologic activity has left specific traces of composition on hydrochemistry, isotopes, and fluid microorganisms, which have significant scientific value in identifying the mechanism of geothermal fluid formation and evolution, leading to a better understanding of deep geothermal resources as well as rational development and use (Amend and Shock, 2001; Inskeep et al., 2010; Vick et al., 2010; Schuler et al., 2017; Wang et al., 2018; Phillips et al., 2021). The deep subsurface environment is usually characterized as an extreme environment with anaerobic conditions, high temperature, high pressure, and high salt, in which microorganisms in the thermal spring build the only ecosystem. The extreme environment and its various geochemical gradients, such as variable ionic strength, wide pH range, and rich mineral elements, have together created a number of stressors in the geothermal setting (Balkwill et al., 2000; Shock et al., 2010; Hou et al., 2013; Inskeep et al., 2013; He et al., 2021). In relatively independent and well-characterized environments, microorganisms form a community structure capable of adaptation that may reflect geoenvironmental characteristics. Non-biological factors, such as tempercature (Sharp et al., 2014; Podar et al., 2020; Zhang S. Q. et al., 2020; Zhang Y. et al., 2020), pH level (Purcell et al., 2007; Boyd et al., 2010; Power et al., 2018), as well as the demand and supply of energy (Kozubal et al., 2008; Schubotz et al., 2013; Beam et al., 2016; Amenabar et al., 2017) (i.e., availability of O_2_ and H_2_S, trace metals and mineral-based electronic receptors and donors) have been the subject of extensive study and verification. Research on thermophilic microorganisms in the hot spring environment was also of great practical importance for the remediation of heavy metals and organic pollution, bioleaching, and the exploration and development of thermoenzymes.

Northwest China has few thermal springs. All of the hot springs in this region are exposed in the peripheries of intrusive rocks and magmatic tectonic zones and propagate in bands. They typically intersect the NW compressional (torsional) fault and the secondary tensional fault of the SN. The hot springs in the research area are controlled by various geological faults and flow paths, which have been shown to produce the “island effect” that drives genetic differentiation (Gaisin et al., 2016; Jungbluth et al., 2016). Most studies of springtime microorganisms have focused on biogeographic patterns of microbial communities in deep-sea magmatic tectonic active zones relative to volcanoes (Miller et al., 2009; Chan et al., 2015; Vishnivetskaya et al., 2015; and others Ng et al., 2005; Lau et al., 2009; Song et al., 2010; Cousins et al., 2018), whereas microorganisms from the hydrothermal environment built for neotectonic movements have not been reported in any detail. For this study, taking the Lower Pleistocene low-temperature thermal storage hot spring (LT) and the Neogene high-temperature hot spring (HT) as examples, here, we report for the first time the bacterial community structure of mid-low-temperature thermal storage in the Gonghe tectonic basin and its correlation with environmental factors, and the distributional characteristics of bacteria in a specific tectonic hot spring environment are explored. Its purpose is to provide a reference and a reasonable guide to excavate the potential ecological, production and use value of its bacterial resources and to promote the protection and reasonable development of hot spring resources.

## 2. Materials and methods

### 2.1. Study region

With a total of 24,400 km^2^ as a land area, Gonghe Basin is the third largest basin in Qinghai Province, and its geographical coordinates are in the range of 98°46′-101°22′E, 35°27′-36°56′N. It is similar in space with a diamond, small in the west and large in the east. The Yellow River flows through the basin on its short axis.

Neotectonic movements are the major internal force for the formation of modern Geomorphy in the field of research. On the one hand, the Gonghe movement of the Early Pleistocene Qinghai-Tibet Movement C episode and the late Middle Pleistocene created features of material composition and spatial structure at the regional scale, thereby constructing a geothermal environment that is unique to the area (Zhang et al., 2004; Yim et al., 2006; Song et al., 2013; Zhang Y. et al., 2020; Zhang et al., 2022). Regional geology and geomorphology have been built up by multiple intermittent and oscillating tectonic uplift movements, and ultimately formed a stratified geomorphic system consisting of the upper plantation system and the lower terrace subsystem, including the foot erosion surface, flood fan surface, basin surface, and the Yellow River terrace surface (Yuan et al., 2013; Craddock et al., 2014; Li et al., 2020; Wang et al., 2020). On the other hand, intra-regional and bounding faults also created conditions necessary for geothermal rock mass uplift and hydrothermal channel building (Supplementary Figure S1).

Under this geological condition, the underground hot water in the study area is mainly hosted in the Lower Pleistocene low-temperature thermal storage and Neogene low-temperature thermal storage (Ma et al., 2020; Zhang S. Q. et al., 2020). The Lower Pleistocene thermal storage cap layer is stable over the 100 to 200 m depth range in the study area. The aquifer consists of subclay and subsandy soils with water temperatures of 18 ~ 42°C and is rich in water. The source of heat for the lake is the Quaternary lower Pleistocene sub clay, sub sandy soil mixed with fine silty sand, medium coarse sand, and gravel containing a thermal reservoir of medium-coarse sand, with a thickness of over 100 m. The water temperature of the Neogene low-temperature medium thermal reservoir can be as high as 40 ~ 85°C. The thermal reservoir is composed of Neogene siltstone, fine sandstone, medium sandstone, and pebbly medium-coarse sandstone, with a buried depth of up to 800–1,150 m. The majority of its heat source is derived from the geothermal convective system of the tectono magmatic belt of the Ela and Waligong mountains (Tang et al., 2020). Therefore, in this study, we distinguish between the two geologic thermal reservoirs with 40°C as the boundary and describe the two thermal reservoir environments with high-temperature hot springs (HT) and low-temperature hot springs (LT) as the code. The low-temperature hot spring temperature is between 21.9°C and 27°C, and the high-temperature hot spring temperature can reach 53.4–83°C.

### 2.2. Sample collection and chemical analysis

**Figure 1:**
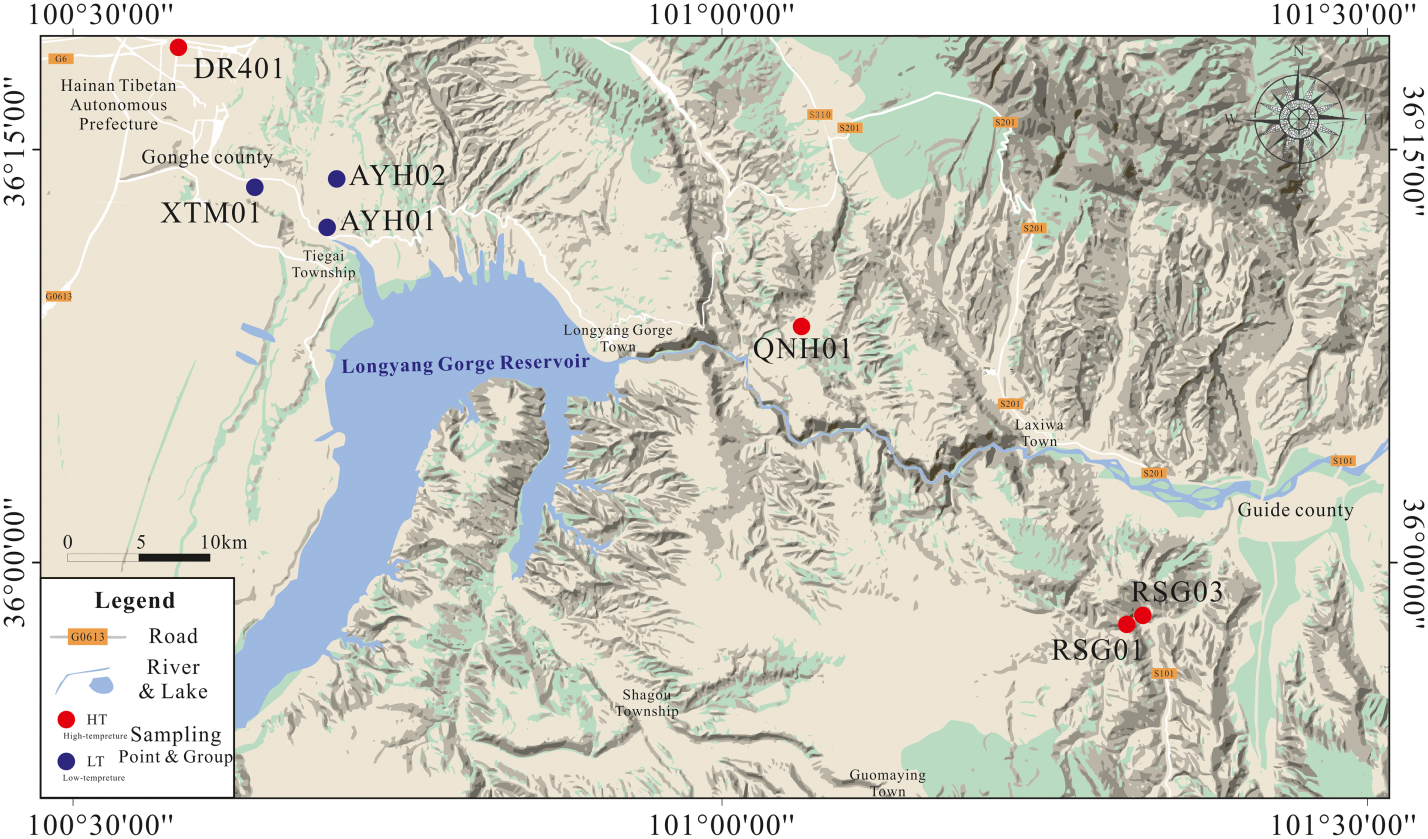
Geographical location and distribution of sample points.

In April 2022, we collected the subsurface hot water samples from 7 exposed hot springs in the thermal reservoirs of Lower Pleistocene and Neogene in the Gonghe Basin for hydrochemical and bacterial analyses (Figure 1). Prior to collecting samples, we observed the outlet of the hot spring until it flowed stably, and then collected samples directly above the submersible pump or at the outlet of the hot spring. DR401 was taken from the geothermal well, and other samples were taken from the outlet of the natural hot spring, with a temperature of 21.9–83°C. Samples for chemical analyses were taken and stored in two 500 ml polyethylene bottles. In the field, all water samples were filtered through 0.45 μm membrane filters to remove particulate debris upon cooling. For analysis of cations, reagent grade HNO_3_ was used to adjust the pH below 2 to prevent oxidation. For analysis of bacteria, biomass was filtered through a sterile, field-preprocessed 0.22 μm membrane and stored in ice boxes, and then transported immediately to the laboratory for further pre-treatment by DNA extraction. Approximately 1 L of springwater was filtered through sterile membranes of 0.22 μm in order to obtain biomass for DNA extraction (Mao et al., 2014; Zhang Y.P. et al., 2018; Guo et al., 2020).

The temperature of the water was measured on site with a digital thermometer to the accuracy of 0.3°C. Total dissolved solids (TDS), pH, electrical conductivity (Ec), redox potential (ORP), and dissolved oxygen (DO) were determined by the Hach HQ40d at the field site (Beijing Hach Instruments Co., Ltd.) with the precision of ±2% full scale for TDS, ±0.01 for pH, ±0.5% full scale for Ec, ±0.1 mV for ORP and ± 0.1 mg/l for DO. The Ca^2+^, Mg^2+^, Na^+^, and K^+^ concentrations were analyzed by Inductively Coupled Plasma Optical Emission Spectrometer (ICP-OES, Thermo Electron Corporation, United States), and H_2_SiO_3_ and other metal elements by Inductively Coupled Plasma Mass Spectrometry (ICP-MS) within 2 weeks after sampling. The CO_3_^2−^ and HCO_3_^−^ concentrations were measured by potentiometric titrations. The SO_4_^2−^ and Cl^−^ concentrations were determined on an unacidified sample according to Ion Chromatography (Dionex-900). Qinghai Geological and Mineral Testing Application Center performed all of the chemical analyses. At the end of the hydrochemical run, we monitored and verified the accuracy of the experimental data based on chemical equilibrium theory (charge balance, precipitation balance, etc.; Garrett and Truhlar, 1980).

### 2.3. DNA extraction and sequencing analysis

Total bacterial genomic DNA samples were extracted using the DNeasy PowerSoil Kit (QIAGEN, Inc., Netherlands), following the manufacturer’s instructions, and stored at −20°C prior to further analysis. The quantity and quality of extracted DNAs were measured using a NanoDrop ND-1000 spectrophotometer (Thermo Fisher Scientific, Waltham, MA, United States) and agarose gel electrophoresis, respectively. PCR amplification of the bacterial 16S rRNA genes V4-V5 region was performed using the forward primer 515F (5’-GTGCCAGCMGCCGCGGTAA-3′) and the reverse primer 907R (5’-CCGTCAATTCMTTTRAGTTT-3′). The PCR components contained 5 μl of Q5 reaction buffer (5×), 5 μl of Q5 High-Fidelity GC buffer (5×), 0.25 μl of Q5 High-Fidelity DNA Polymerase (5 U/μl), 2 μl (2.5 mM) of dNTPs, 1 μl (10 uM) of each Forward and Reverse primer, 2 μl of DNA Template, and 8.75 μl of ddH_2_O. Thermal cycling consisted of initial denaturation at 98°C for 2 min, followed by 25 cycles consisting of denaturation at 98°C for 15 s, annealing at 55°C for 30 s, and extension at 72°C for 30 s, with a final extension of 5 min at 72°C. PCR amplicons were purified with Agencourt AMPure Beads (Beckman Coulter, Indianapolis, IN) and quantified using the PicoGreen dsDNA Assay Kit (Invitrogen, Carlsbad, CA, United States). After the individual quantification step above, amplicons were pooled in equal amounts, and paired-end 2 × 300 bp sequencing was performed using the Illlumina MiSeq platform with MiSeq Reagent Kit v3 at Shanghai Personal Biotechnology Co., Ltd., (Shanghai, China). The raw reads were submitted into the NCBI Sequence Read Archive (SRA) database (accession number: SRP389411).

### 2.4. Bioinformatics and statistical analysis

Microbiome bioinformatics were performed using QIIME2 2019.4 with slight modification according to the official tutorials. Briefly, raw sequence data were demultiplexed using the demux plugin following by primers cutting with cutadapt plugin. Sequences were then quality filtered, denoised, merged and chimera removed using the DADA2 plugin. The ASV-table was transformed using the method of rarefaction by using the qiime feature table rarify function of QIIME2 software in the data standardization process, the flattening depth was set to 95% of the lowest sample sequence size, so that samples could be analyzed at the same sequencing depth level. Alpha-diversity metrics (Chao1, Observed species, Shannon, Simpson, Faith’s PD, Pielou’s evenness and Good’s coverage), beta diversity metrics, unweighted UniFrac, Jaccard distance, and Bray–Curtis dissimilarity were estimated using the diversity plugin with samples were rarefied to 51,058 sequences per sample. Taxonomy was assigned to ASVs using the classify-sklearn naïve Bayes taxonomy classifier in feature-classifier plugin against the SILVA Release 132 Database.

Sequence data analyses were mainly performed using QIIME2 and R packages (v3.2.0). ASV-level alpha diversity indices, such as Chao1 richness estimator, Observed species, Shannon diversity index, Simpson index, Faith’s PD, Pielou’s evenness and Good’s coverage were calculated using the ASV table in QIIME2, and visualized as box plots. ASV-level ranked abundance curves were generated to compare the richness and evenness of ASVs among samples. Beta diversity analysis was performed to investigate the structural variation of bacterial communities across samples using unweighted pair-group method with arithmetic means (UPGMA) hierarchical clustering. Principal component analysis (PCA) was also conducted based on the ASV-level compositional profiles. A Venn diagram was generated to visualize the shared and unique ASVs among samples or groups using R package “Venn Diagram,” based on the occurrence of ASVs across samples/groups regardless of their relative abundance. Random forest analysis was applied to discriminating the samples from different groups using QIIME2 with default settings.

Spatial distribution characteristics were analysed using Mapgis software. Data collection and statistical analyses were performed using Excel 2007, SPSS 25.0, and Canoco 5.

## 3. Analysis and discussion

### 3.1. Hydrochemical characteristics

In the study area, subsurface samples from hot springs had pH values of 7.23 to 7.96 (Supplementary Table S1). The mean temperature difference between the two heat reservoirs was as high as 44.45°C, and the ORP level was also significantly different (−141.2 ~ −247.8 mv), representing the environment of hydrothermal occurrence with different characteristics of the environment. With respect to the water chemistry index, K^+^, SO_4_^2−^, and NO_3_^−^ were higher in the HT group but Mg^2+^ and HCO3^−^ were lower, whereas SO_4_-Cl·Na water was the major water type overall (Supplementary Figure S2). One of the possible sources of SO_4_^2−^ in geothermal water in the study area was the dissolution of sulfate minerals (e.g., Gypsum) along the seepage pathway, and it may also be influenced by geochemical processes, such as the oxidation of pyrite in the rock media at the same time (Dillon et al., 2007).

From a regional geologic perspective, the water samples collected in this study are primarily concentrated in the Gonghe Basin region and two near-SN trending areas of tectonic-magmatic uplift on its east and west sides. As HT group samples are from the deep part, the hot water cycle is long, the run-off path is distant, the interaction between the water body and the surrounding environment is strong, and the content of certain chemical constituents of water in the geothermal fluid is relatively high (Zhang C. et al., 2018). Notably, it is also reflected in the H_2_SiO_3_, F^−^, and other indicators. Calculation data from earlier quartz thermometers show that Lower Pleistocene thermal storage belongs to a typical mid to low-temperature intraplate geothermal system (Ma et al., 2020). There is little water-rock interaction and it is possible that the geothermal fluid is not affected by magmatic water. As a result, it exhibits the above features. It is evident that the hydrochemical composition of geothermal water is affected by multiple geochemical processes, such as the dissolution of gypsum and oxidation of minerals.

### 3.2. Community structure of bacteria

#### 3.2.1. Diversity analysis

**Figure 2:**
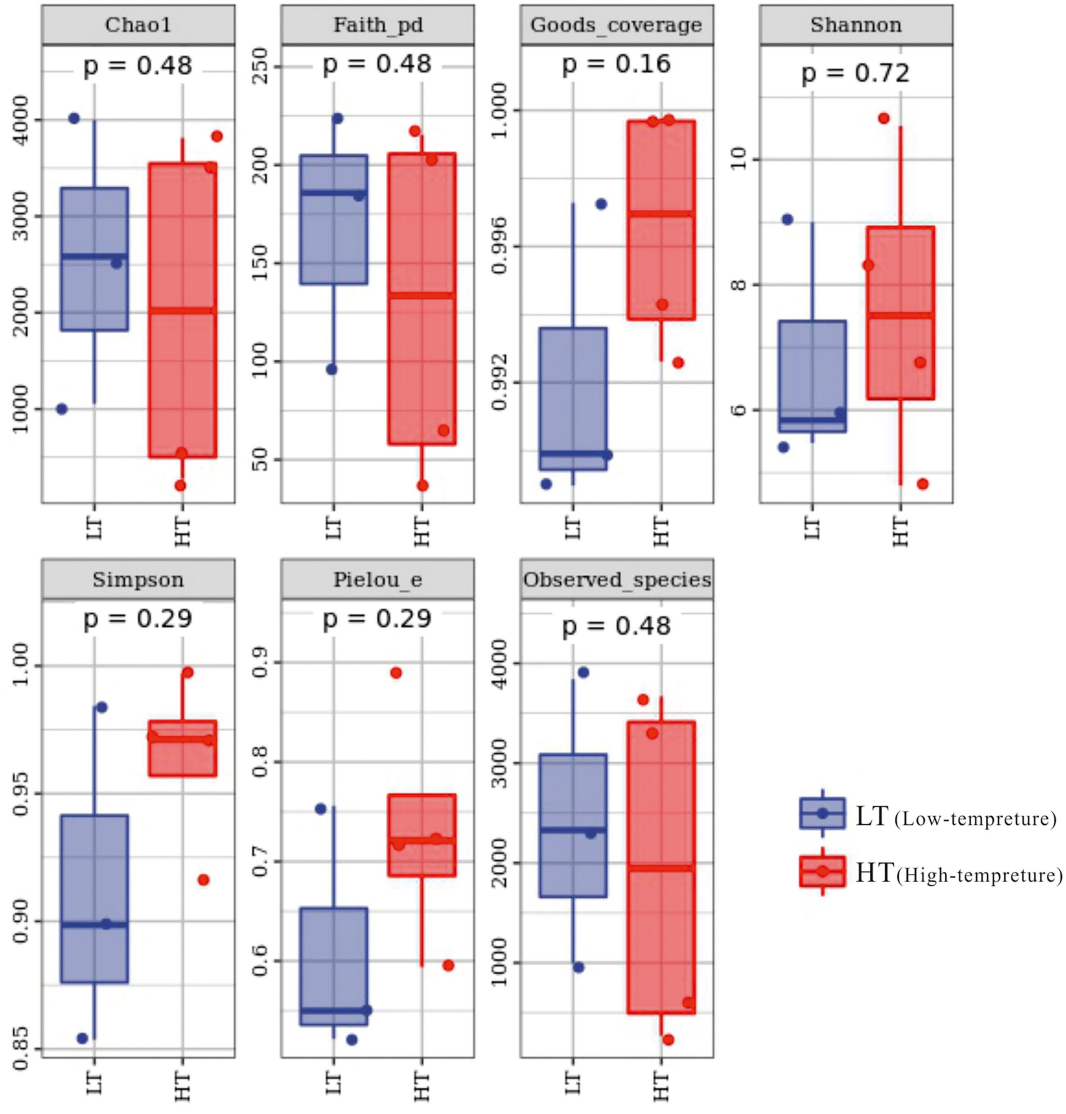
Box chart of Alpha index of groundwater bacterial community.

The total amount of sequences obtained in the study was 130,303 ~ 147,201 pairs, and 59,186 ~ 120,716 high-quality sequence sets were analyzed and tested. The coverage index varied from 0.989 to 0.9998 in the sequenced samples, embodying a high and reliable depth of sequencing, as can be seen from the alpha index boxplots (Figure 2). In terms of Chao1 index, the LT cluster had an means of 2545.887, which was slightly greater than the means for the HT group of 2031.096, which together with the Observed_species and Faith_pd indices reflected that samples from the LT group had relatively high richness and were superior in diversity based on the evolution (Supplementary Table S1).

#### 3.2.2. Bacterial composition analysis

**Figure 3:**
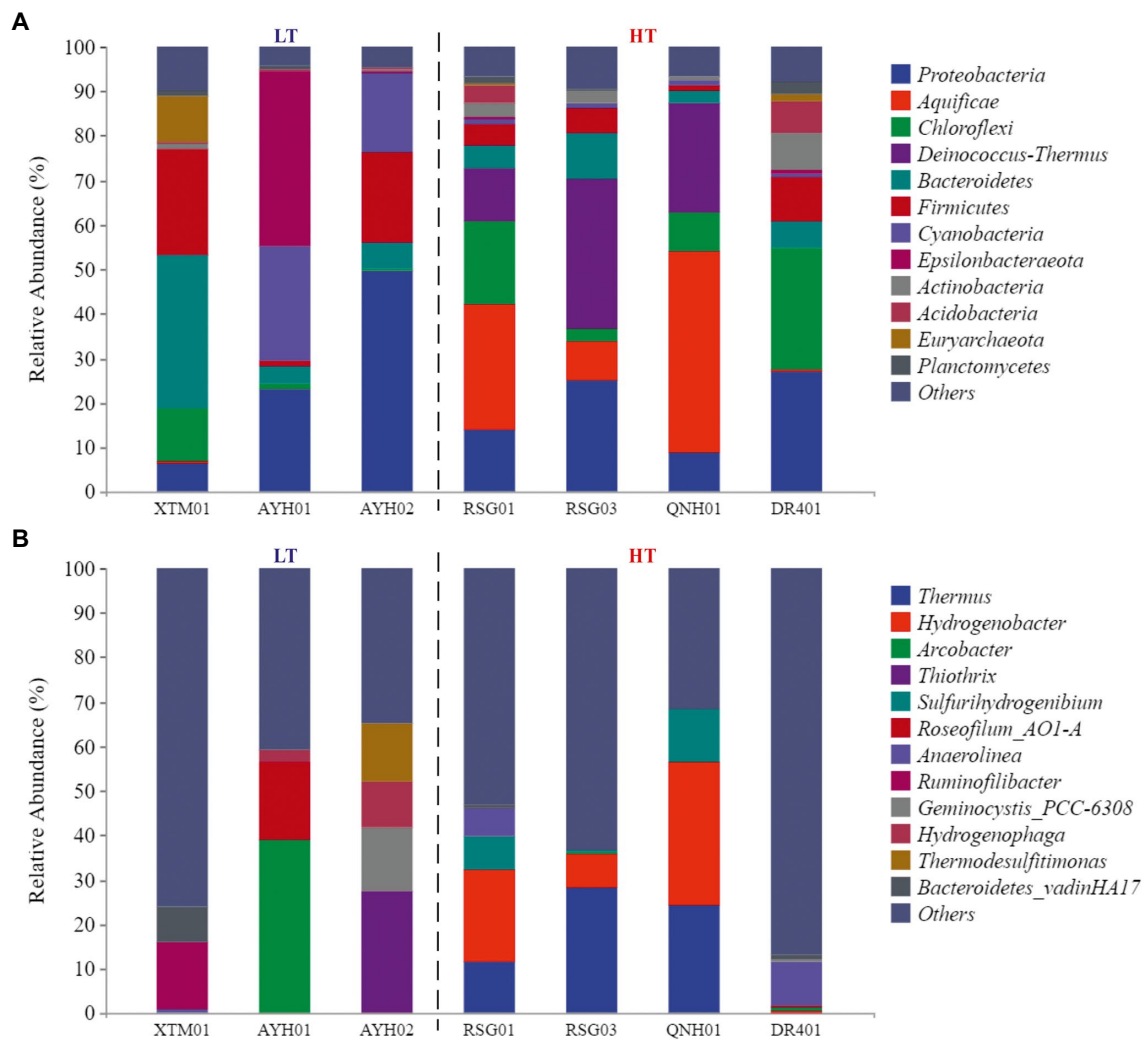
The phylum composition **(A)** and genus composition **(B)** of the groundwater bacterial community.

At the phylum level, the species composition differed among the groups, with *Proteobacteria* (21.99%), *Chloroflexi* (10.14%), *Bacteroidetes* (9.77%) and *Firmicutes* (9.61%) more abundant and stable species at the phylum level (Figure 3A). On the one hand, the species with the highest levels of abundance and greater than 10% in the LT group were *Proteobacteria* (26.41%), *Firmicutes* (15.17%), *Bacteroidetes* (14.64%), *Cyanobacteria* (14.55%), *Epsilonbacteraeota* (13.20%). On the other hand, in the HT group, they were *Aquificae* (20.72%), *Proteobacteria* (18.68%), *Deinococcus*-*Thermus* (17.48%), and *Chloroflexi* (14.31%). It can be seen that *Proteobacteria*, *Bacteroidetes*, and *Firmicutes* were the dominant species with the most robust statuses in both environments. *Proteobacteria* was the largest phylum of bacteria and was extremely rich in species diversity and genetic diversity, and studies had discovered its dominance in both ultra-high-temperature hot springs and in general-temperature hot springs (Zhang et al., 2018a). *Firmicutes* had high amounts of peptidoglycan in their cell walls, were predominantly globular or rod-shaped, and were able to produce endospores that were resistant to dehydration and extreme environments (Zhao et al., 2021). Both species were extremely adaptable in hot spring habitats.

*Cyanobacteria* (+13.56%) and *Epsilonbacteraeota* (+12.87%) were almost exclusively found in low-temperature environments, while *Aquificae* (+20.7%) and *Deinococcus*-*Thermus* (+17.42%) were almost exclusively found in high-temperature hot spring environments. Research conducted in Japan, New Zealand, Italy, and Africa by Papke et al. (2003) has confirmed that the maximum suitable *Cyanobacteria* temperature in the geologic hot-spring environment is 63°C. *Cyanobacteria* from warm springs had smaller genomes, higher GC content, shorter proteins, and were more hydrophilic and alkaline than those from nonthermal sources (Alcorta et al., 2020). Comparative genomics studies of *Cyanobacteria* have revealed the genetic capacity for oxygenic photosynthesis that utilizes photosystems I and II in heat storage, and oxygenic photosynthesis that utilizes a putative sulfide-quinone reductase to oxidize the sulfide and bypasses photosystem II (Lily et al., 2019). In the case of high-temperature environment species, Tamazawa et al. (2016) reported that *Aquificae* could grow chemolithoautotrophically within the primary geologic environment and played essential roles as a primary producer within the sulfinic hot-spring ecosystem. *Aquificae* was a phylum of thermophilic to hyperthermophilic chemolithoautotrophic bacteria, in which the gene for nitrogenase reductase (nifH) has been confirmed to exist (Wirth et al., 2010; Zeytun et al., 2011). Both strains were assayed for nitrogenase activity at 70°C. Nishihara et al. (2018) used acetylene reduction to confirm that the phylum *Aquificae* had a nitrogen-fixing function. A few new strains of *Aquificae* were known to grow under semi-aerobic conditions using CO_2_ as the sole carbon source and N_2_ as the sole nitrogen source in media containing hydrogen and/or thiosulfate.

At the genus level, the different samples in the LT group were more diverse in composition and did not have any dominant genera (Figure 3B). Within the HT group, *Thermus* (15.96%) and *Hydrogenobacter* (15.19%) were the dominant genera in the samples collected. *Thermus* was found in both shallow-ocean and deep-ocean hydrothermal systems as well as low-salinity insolation hot springs, where it has been shown to adapt to temperature (55–100°C) and pH interval (pH 5–9). Originally isolated from warm spring environments, *Hydrogenobacter* was an autotrophic, extremely thermophilic, hydrogen-oxidizing bacteria with an optimal growth temperature of approximately 70–75°C (Zhang et al., 2018b).

#### 3.2.3. Analysis of community differences in bacterial composition

As can be observed in Supplementary Figure S3, there was a considerable difference in ASVs composition of microbes from sub-surface hot springs between the HT group and the LT group. Only 195 ASVs were shared between the HT group and the subsurface hot spring in the LT group, among which 6,905 unique ASVs were found in the LT group, while 6,722 were found in the HT group (Supplementary Figure S3). As can be seen, the geologic environment represented by the different sample groups exerted a profound influence on the ASV community structure. Except for temperature and redox environment, other hydrochemical traits or hydrologic processes may dominate the survival of some of the colonies.

At the phylum level, there were 17 geothermal hot spring species in the study region, including 8 species with an abundance level greater than 2% (Supplementary Figure S4). Within the major genera, *Deinococcus*-*Thermus* included some spherical bacteria that were able to withstand harsh environments, including Anococcus and Thermophila. The genus Anococcus includes multiple radiation-resistant bacterial species, which have been shown to have degradative effects on nuclear and other toxic substances (Li et al., 2017). *Thermophila* included many thermotolerant genera, among which thermotolerant DNA polymerase (Taq enzyme) isolated from *Thermous aquaticus* was widely used in polymerase chain reactions (Bakovic et al., 2021). In many extreme environments, such as deserts, hot springs, and high-salt environments, *Cyanobacteria* was a locally dominant producer. In hot springs, one of the thermophilic *Cyanobacteria* was able to reach a temperature of 73°C, achieving the upper limit of phototrophic temperature (Otaki et al., 2012). Furthermore, at the phylum level, *Thaumarchaeota* and GAL15 were found to be endemic to the HT group. *Thaumarchaeota* performed autotrophic metabolism and mixotrophic growth in environments, such as marine, soil, and freshwater sediments, and were widely involved in denitrification (Brochier-Armanet et al., 2008; Zhang and He, 2012).

**Figure 4:**
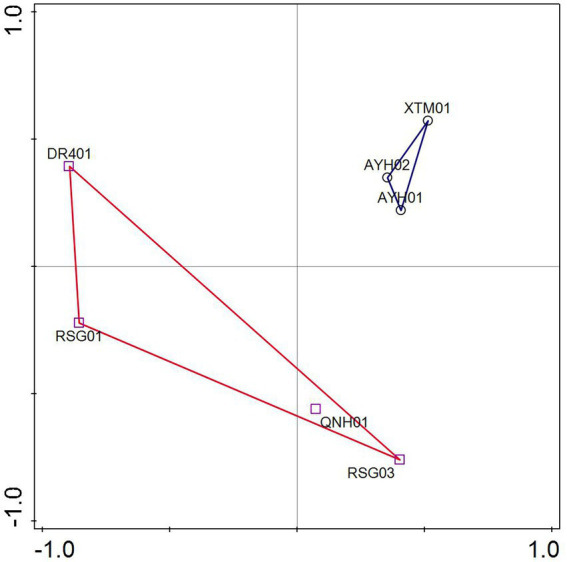
Principal coordinate analysis of samples.

ASVs were analyzed by PCA based on the species matrix. The variation characteristics of 32.22 and 22.71% for PC1 and PC2, respectively, were shown in Figure 4. The LT cluster was primarily distributed in the 0.3 ~ 0.55 positive PC1 axis, whereas the HT group was spread in the region between −1 and 0.5. The two groups appeared to have an intuitive distribution, which indicated that species with significant environmental features can be distinguished at the ASV level.

**Figure 5:**
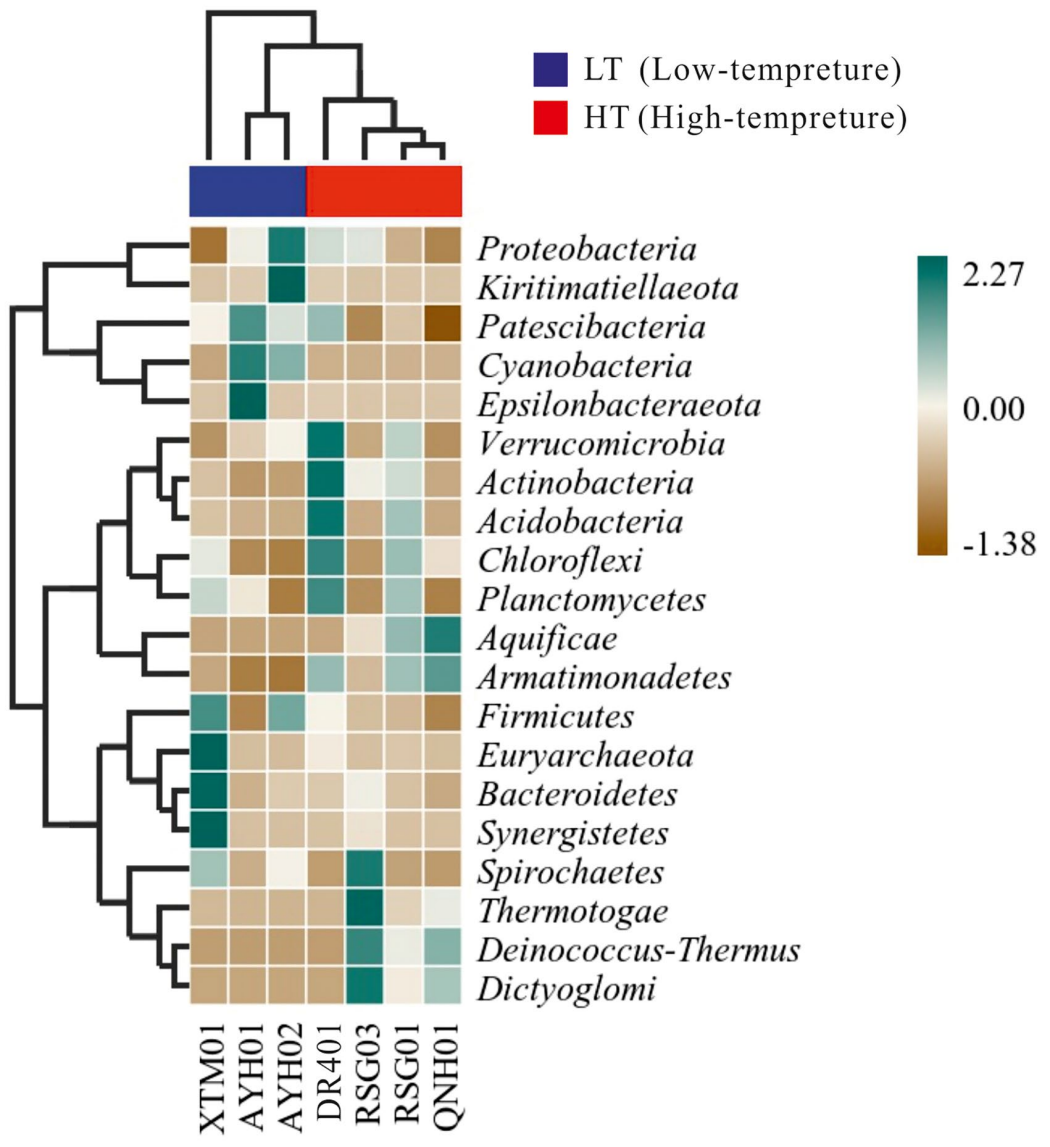
Heatmap of the bacterial phylum in hot springs on the Northeast Tibetan Plateau.

We conducted distance-based redundancy analysis (db-RDA) to determine if environmental drivers drived bacterial community structure with a constrained ordination method. Constrained ordination techniques attempted to account for differences in microbial composition among samples through differences in explanatory variables. Factors related to the water environment (i.e., T, ORP, *p* = 0.034) exhibited a relationship with the structure of the bacterial community (Supplementary Figure S5). As microbial community characteristic data often exhibited a discontinuous distribution or even discrete features, a heat map and a random forest analysis established a nonlinear decision tree-based sample classifier for searching for marker species, which was used extensively in the analysis of environmental microbes (Menzel et al., 2015; Richard et al., 2017). It can be seen from the thermal map of community composition at the phylum level that Verrucomicrobia, Actinobacteria, Acidobacteria, and Armatimonadetes were more abundant in the HF group, while Cyanobacteria, Firmicutes were more indicative in the LT group (Figure 5).

*Acidobacteria* was a large phylum of bacteria but due to their limited culturability for them at this stage, his native environment was difficult to simulate accurately, limiting the scope of his research. This is the case for *Verrucomicrobia* as well (Kielak, 2010). *Actinobacteria* were able to survive in different environments due to their ability to produce a variety of extracellular hydrolases, especially in soils, which were responsible for the decomposition of a dead plant, animal, and fungal matter, making them a central species in the carbon cycle (Mohammadipanah and Wink, 2016).

**Figure 6:**
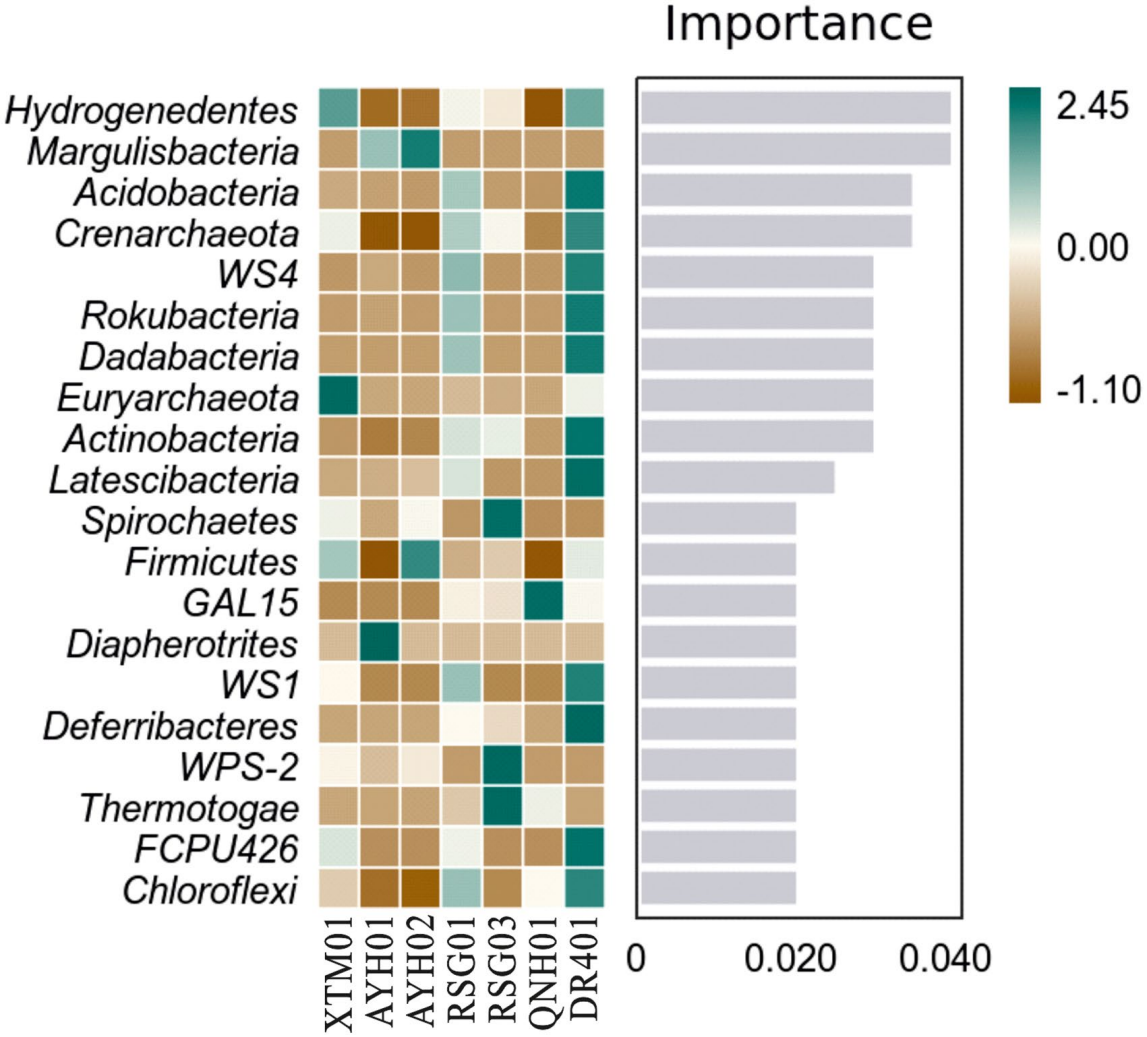
Random forest analysis of bacterial phyla in hot springs on the Northeast Tibetan Plateau.

Random forest analysis showed that the phyla *Hydrogenedentes*, *Margulisbacteria*, and *Acidobacteria* were the marker species for the differences between the groups as computed by the classifier model (Figure 6). *Hydrogenedentes* was one such class of autotrophic microorganism that can generate energy by oxidizing H_2_. *Margulisbacteria* was often found in symbiosis with other species and can use fats and other lipids to supply vitamins and amino acids to commensal species. They were considered to be strict anaerobes based on the absence of cytochrome oxidase, catalase, and superoxide dismutase, and the presence of rubrerythrin and rubredoxin can facilitate the survival of these bacteria during times of oxidative stress (Lumppio et al., 2001). Based on species traits, we believed that temperature, reduced environmental intensity, and certain hydrogeochemical processes were more important drivers of community traits and intergroup differences.

### 3.3. Analysis of correlations between bacterial community and environmental traits

Concerning hydrologic characteristics, bacterial population abundance was significantly correlated with certain hydrologic characteristics. *Aquificae* and *Deococcus*-*Thermus* preferred a high-temperature environment with a strong water-rock interaction; *Chloroflexi*, *Actinobacteria*, *Acidobacteria* preferred a low-mineralization environment and a high-temperature NO3- rich environment; Bacteroidetes and Firmicutes preferred low and moderate temperature environment with weak alkalinity and reductivity; *Cyanobacteria* and *Epsilonbacteraeota* preferred a medium-and low-temperature environment rich in nitrogen and DO. At the genus Thermous level, the extreme thermophilic bacteria exhibited the broad aerobic properties that were shared with *Thermous*, but isolates of this species were found to grow anaerobically with nitrate as a substrate in the first step of the denitrification pathway, and generate nitrite as an end-product (Ramirez-Arcos et al., 1998). Other species in the Thermous genus were even capable of complete denitrification, the reduction of nitrite to N_2_ as the end product (Rainey and da Costa, 2001). Some strains of *T. thermophilus* were capable of using nitrate as an electron acceptor during anaerobic growth, and this required the aerobic respiratory chain to be replaced, whose main electron donor was the Nqo type I NDH, by a specific respiratory chain made of the hetero tetrameric enzymes NDH (Nrc) and nitrate reductase (Nar) (Cava et al., 2004, 2007).

As can be seen, the water environment built by geologic temperature and water-rock interaction has been the central factor determining the microbial community structure of hot springs (Kozubal et al., 2008; Cole et al., 2013; Wang et al., 2013; Sharp et al., 2014; Fortney et al., 2016; Liu et al., 2017, 2018). The degree of sealing and metamorphism of the formation water can be indicated by the characteristic coefficient calculated from the hydrochemical data of the subsurface hot water (Table 1). As can be seen, the metamorphic coefficient ratio (*r*Na^+^/*r*Cl^−^) ranged from 1.19 to 2.27, higher than the threshold value of water body seep activity intensity of 0.85, indicating that seepage water activity was intense and formation water tightness and metamorphism were less than ideal. The desulfurization coefficient (100*rSO_4_^2−^/*r*Cl^−^) was found to be 12–74.67, which was larger than the threshold value of desulfurization intensity of 1. This indicated that the environment had poor sealing conditions, a low organic content, incomplete reduction, and was commonly affected by oxidation from the outside. Of these, AYH01 and QNH01 had the lowest desulfurization coefficient, and the H2S odor could clearly be smelled during the sampling process. The salinity coefficient (*r*Cl^−^/*r*HCO_3_^−^ + *r*CO_3_^2−^) represented the water concentration of the formation. Given the values and spatial conditions, the salinity coefficient increased gradually from the north to the south of the study area, fundamentally in accordance with the pattern of change in mineralization. Guo et al. (2020) similarly suggested that microbial communities of hot springs were more related to environmental characteristics than the predominant geographic distribution, and filtering of the environment on the microbial community was greater than the geographical distribution of microorganisms in those environments.

**Table 1:** The characteristic hydrochemical coefficient of thermal storage of hot springs in the northeast margin of the Qinghai Tibet plateau.

		Metamorphism coefficient (*r*Na^+^/*r*Cl^−^)	Desulfurization coefficient (100*rSO_4_^2−^/*r*Cl^−^)	Salinization coefficient (*r*Cl^−^/*r*HCO_3_^−^ + *r*CO_3_^2−^)
LT (*n* = 3)	XTM01	1.7291	46.2006	2.5613
	AYH01	1.1913	12.0067	7.7420
	AYH02	2.2693	65.9968	1.8711
	Avg	1.7299	41.4014	4.0581
HT (*n* = 4)	DR401	1.8813	33.5921	0.8226
	RSG01	2.0703	74.6669	9.8951
	RSG03	2.0219	72.5311	9.6259
	QNH01	1.3128	21.4244	18.6780
	Avg	1.8215	50.5536	9.7554

**Figure 7:**
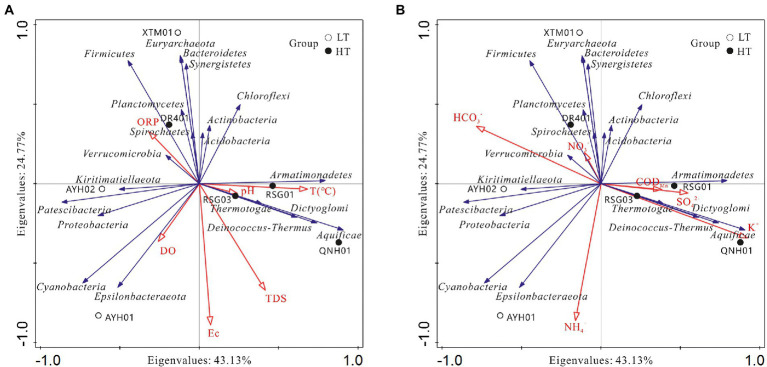
Analysis of relationships among samples, influencing factors，and bacterial phyla. **(A)** Hydrological factor; **(B)** hydrochemical factor.

To visually demonstrate the relationship between each species and the factors of hydrographic and water chemistry traits, we selected key indicators of environmental characteristics and analyzed the species in the top 20 positions at the phylum level and abundances across samples for redundancy in RDA (Figure 7).

As can be seen, the species were associated with the hydrochemical indicators and formed relatively independent response clusters: (1) The abundances of *Aquificae*, *Deinococcus*-*Thermus*, *Armatimonadetes,* and *Dictyoglomi* were positively correlated with temperature, K^+^, SO_4_^2−^ and H_2_SiO_3_, which reached extremely significant levels with K^+^ and H_2_SiO_3_, and were negatively correlated with Mg^2+^ and HCO_3_^−^. These taxa were found in such extreme environments as hot springs, sulfur pools, and the mouths of hot springs on seamounts and can survive and thrive in environments as high as 95°C. In addition, this extreme group of thermophilic bacteria was industrially valuable, such as *Dictyoglomus*, which was able to use organic matter for energy and could produce xylanase and thereby break down xylan (Peacock et al., 2013). (2) *Chloroflexi*, *Actinobacteria*, *Acidobacteria*, and *Verrucomicrobia* were found to be positively correlated with temperature, COD_Mn_, NO_3_^−^, and negatively correlated with TDS and Na^+^. The case of *Acidobacteria* and *Verrucomicrobia* has been discussed above. (3) *Bacteroidetes*, *Firmicutes*, *Euryarchaeota*, and *Synergistetes* were found to be positively correlated with Ca^2+^, ORP, HCO_3_^−^, and Br^−^. The genus *Euryarchaeota* was diverse and had numerous functional traits and branches, including halophilic, thermophilic, and methanogenic. *Synergistetes* have been used in a similar manner for the industrial degradation of organic compounds (Boyd et al., 2009; Schuler et al., 2017). In a functional sense, this group could be considered a core species for carbon cycling processes in hot spring environments. (4) *Cyanobacteria* and *Epsilonbacteraeota* were positively correlated with DO, Na^+^, Cl^−^, NO_2_^−^ and NH_4_^+^, which have been shown to reach significance with NO_2_^−^ and NH_4_^+^, presumably important species involved in the nitrogen cycling of hydrothermal ecosystems.

In this study, we combined bacterial groups and their relationship with environmental factors and found that, in many major bacterial populations, the abundances of unique species in the second and third clusters were all positively correlated with Chao1, Simpson, Shannon, Faith_pd, and Pielou_e, and the positive correlation between cluster 2 and the Simpson index and Pielou_e reached significance (Supplementary Table S2). Therefore, we believed both of these groups to be producers of hydrothermal ecosystems, and there were differences in resource use between them as described previously. Furthermore, as a species capable of utilizing NO_3_^−^, *Planctomycetes* had a highly significant positive correlation with Chao1 and Simpson indices, and it has also been one of the major producers of hydrothermal ecosystems. As can be seen, bacterial population abundances and environmental indicators exhibited their respective correlations, indicating that the majority of bacterial populations were susceptible to and exhibited a relationship with the hydrochemical process. The hydraulic environment constructed jointly by geologic temperature and water-rock interaction was the central factor determining the bacterial community structure of the hot springs.

## 4. Conclusion

In this study, we investigated the key driving forces affecting the composition and distribution of bacterial communities in hot springs in two geologic thermal reservoirs located within a low-temperature convection-type geothermal system in magmatic tectonic zones of the Northeast Tibetan Plateau. The results of this study may further our understanding of the bacterial distribution and biogeochemical cycling in geothermal reservoirs with different properties, and may also have implications for other similar environments. The characteristics of the chemical composition of the water and the thermal storage environment of the low-temperature convection-type geothermal hot springs in the study area were different, which were influenced by numerous geochemical processes, such as the dissolution of gypsum and oxidation of minerals. The bacterial community structures in the two hot spring tanks were very different. Low-temperature thermal springs in the Lower Pleistocene had a uniform species composition, such as *Arcobacter*, *Thiothrix*, and *Roseofilum*, whereas high-temperature Neogene thermal springs were dominated by typical thermophilic microorganisms, such as *Thermus* and *Hydrogenobacter*. Relative abundance levels of the subsurface hot spring in the study area were found to be dependent on the elevated temperature and mildly alkaline reducing environment. Differences between the groups were primarily due to temperature, the intensity of the reducing environment, and certain hydrogeochemical processes. The geological environment represented by the different pooled samples exerted profound effects on the structure of the bacterial community, of which temperature and redox potential were the most important factors.

## Data availability statement

The datasets presented in this study can be found in online repositories. The names of the repository/repositories and accession number(s) can be found below: NCBI, PRJNA860942.

## Author contributions

H-sZ: put forward the design plan of this research, presided over and participated in all stages of work, and independently completed the writing of this paper. Q-dF: provided the conditions and source of fund for field investigation, participated in data collection and experiment design, and mainly engaged in the analysis and research of the background of the research area. D-yZ: participated in the experiment design and data analysis, and assisted in the modification and production of some sketches. G-lZ: participated in data collection, data processing and technical support of water chemistry. LY: participated in the research on data collection, environment factors and the Assisted Research on the process of water and earth chemistry.

## Funding

The Geological survey project of China Geological Survey (grant: DD20211336)， open fund of State Key Laboratory of Biogeography and Environmental Geology (grant: GBL22110).

## Conflict of interest

The authors declare that the research was conducted in the absence of any commercial or financial relationships that could be construed as a potential conflict of interest.

## Publisher’s note

All claims expressed in this article are solely those of the authors and do not necessarily represent those of their affiliated organizations, or those of the publisher, the editors and the reviewers. Any product that may be evaluated in this article, or claim that may be made by its manufacturer, is not guaranteed or endorsed by the publisher.

## References

[ref02] Alcorta J. Alarcón-Schumacher T. Salgado O. Díez B. (2020). Taxonomic Novelty and Distinctive Genomic Features of Hot Spring Cyanobacteria. Front. Genet. 11:568223. doi: 10.3389/fgene.2020.568223 33250920 PMC7674949

[ref01] Amenabar M. J. Shock E. L. Roden E. E. Peters J. W. Boyd E. S. (2017). Microbial substrate preference dictated by energy demand rather than supply. Nat. Geosci. 10, 577–583. doi: 10.1038/Ngeo2978 30944580 PMC6443248

[ref1] Amend J. P. Shock E. L. (2001). Energetics of overall metabolic reactions of thermophilic and hyperthermophilic archaea and bacteria. FEMS Microbiol. Rev. 25, 175–243. doi: 10.1111/j.1574-6976.2001.tb00576.x, PMID: 11250035

[ref2] Bakovic V. Martin Cerezo M. L. Höglund A. Fogelholm J. Henriksen R. Hargeby A.. (2021). The genomics of phenotypically differentiated Asellus aquaticus cave, surface stream and lake ecotypes. Mol. Ecol. 30, 3530–3547. doi: 10.1111/mec.15987, PMID: 34002902

[ref3] Balkwill D. L. Kieft T. L. Tsukuda T. Kostandarithes H. M. Onstott T. C. Macnaughton S.. (2000). Thermus multireducens sp. nov., a globally distributed metal-reducing species associated with thermal ground and spring waters.

[ref4] Beam J. P. Bernstein H. C. Jay Z. J. Kozubal M. A. Jennings R. D. Tringe S. G.. (2016). Assembly and succession of iron oxide microbial mat communities in acidic geothermal springs. Front. Microbiol. 7:25. doi: 10.3389/fmicb.2016.00025, PMID: 26913020 PMC4753309

[ref5] Boyd E. S. Hamilton T. L. Spear J. R. Lavin M. Peters J. W. (2010). [FeII]-hydrogenase in Yellowstone National Park: evidence for dispersal limitation and phylogenetic niche conservatism. ISME J. 4, 1485–1495. doi: 10.1038/ismej.2010.76, PMID: 20535223

[ref6] Boyd E. S. Leavitt W. D. Geesey G. G. (2009). CO_2_ uptake and fixation by a thermoacidophilic microbial community attached to precipitated sulfur in a geothermal spring. Appl. Environ. Microbiol. 75, 4289–4296. doi: 10.1128/AEM.02751-08, PMID: 19429558 PMC2704841

[ref7] Brochier-Armanet C. Boussau B. Gribaldo S. Forterre P. (2008). Mesophilic Crenarchaeota: proposal for a third archaeal phylum, the *Thaumarchaeota*. Nat. Rev. Microbiol. 6, 245–252. doi: 10.1038/nrmicro1852, PMID: 18274537

[ref8] Cava F. Laptenko O. Borukhov S. Chahlafi Z. Blas-Galindo E. Gómez-Puertas P.. (2007). Control of the respiratory metabolism of *Thermus thermophilus* by the nitrate respiration conjugative element. NCE 64, 630–646. doi: 10.1111/j.1365-2958.2007.05687.x, PMID: 17462013

[ref9] Cava F. Zafra O. Magalon A. Blasco F. Berenguer J. (2004). A new type of NADH dehydrogenase specific for nitrate respiration in the extreme thermophile *Thermus thermophilus*. J. Biol. Chem. 279, 45369–45378. doi: 10.1074/jbc.M404785200, PMID: 15292214

[ref10] Chan C. S. Chan K. G. Tay Y. L. Chua Y. H. Goh K. M. (2015). Diversity of thermophiles in a Malaysian hot spring determined using 16S rRNA and shotgun metagenome sequencing. Front. Microbiol. 6:177. doi: 10.3389/fmicb.2015.00177, PMID: 25798135 PMC4350410

[ref12] Cole J. K. Peacock J. P. Dodsworth J. A. Williams A. J. Thompson D. B. Dong H.. (2013). Sediment microbial communities in great boiling spring are controlled by temperature and distinct from water communities. ISME J. 7, 718–729. doi: 10.1038/ismej.2012.157, PMID: 23235293 PMC3605714

[ref13] Cousins C. R. Fogel M. Bowden R. Crawford I. Boyce A. Cockell C.. (2018). Biogeochemical probing of microbialcommunities in a basalt-hosted hot spring at Kverkfjöllvolcano, Iceland. Geobiology 16, 507–521. doi: 10.1111/gbi.12291, PMID: 29856116

[ref14] Cox S. C. Menzies C. D. Sutherland R. Denys P. H. Chamberlain C. Teagle D. A. H. (2015). Changes in hot spring temperature and hydrogeology of the alpine fault hanging wall, New Zealand, induced by distal South Island earthquakes. Geofluids 15, 216–239. doi: 10.1111/gfl.12093

[ref15] Craddock W. H. Kirby E. Zhang H. P. Clark M. K. Champagnac J. D. Yuan D. (2014). Rates and style of Cenozoic deformation around the Gonghe Basin, northeastern Tibetan plateau. Geosphere 10, 1255–1282. doi: 10.1130/GES01024.1

[ref16] Deng J. Xiao C. Wang Q. Zhou X. Yang L. Zhang J.. (2010). Influence of the Chuxiong Yao’an earthquake on the mineralization of hot springs in the Tengchong geothermal area, southwestern China. Acta Geol. Sin-Engl. 84, 345–357. doi: 10.1111/j.1755-6724.2010.00148.x

[ref17] Dillon J. G. Fishbain S. Miller S. R. Bebout B. M. Habicht K. S. Webb S. M.. (2007). High rates of sulfate reduction in a low-sulfate hot spring microbial mat are driven by a low level of diversity of sulfate-respiring microorganisms. Appl. Environ. Microbiol. 73, 5218–5226. doi: 10.1128/AEM.00357-07, PMID: 17575000 PMC1950965

[ref18] Fortney N. W. He S. Converse B. J. Beard B. L. Johnson C. M. Boyd E. S.. (2016). Microbial Fe(III) oxide reduction potential in chocolate pots hot spring, Yellowstone National Park. Geobiology 14, 255–275. doi: 10.1111/gbi.12173, PMID: 26750514

[ref19] Gaisin V. A. Grouzdev D. S. Namsaraev Z. B. Sukhacheva M. V. Gorlenko V. M. Kuznetsov B. B. (2016). Biogeography of thermophilic phototrophic bacteria belonging to Roseiexusgenus. FEMS Microbiol. Ecol. 92, 1–17. doi: 10.1093/femsec/fiw012, PMID: 26826142

[ref03] Gao J. Zhang H. J. Zhang S. Q. (2018). Three-dimensional magnetotelluric imagine of the geothermal system beneath the Gonghe basin. Northeast Tibetan Plateau, Geothermic 76, 15–25. doi: 10.1016/j.geothermics.2018.06.009

[ref20] Garrett B. C. Truhlar D. G. (1980). Improved canonical variational theory for chemical reaction rates. Classical mechanical theory and applications to collinear reactions. J. Phys. Chem. 84, 805–812. doi: 10.1021/j100444a020

[ref21] Guo L. Wang G. Sheng Y. Sun X. Shi Z. Xu Q.. (2020). Temperature governs the distribution of hot spring microbial community in three hydrothermal fields, eastern Tibetan plateau Geothermal Belt, Western China. Sci. Total Environ. 720:137574. doi: 10.1016/j.scitotenv.2020.137574, PMID: 32145630

[ref22] He Q. Wang S. Hou W. Feng K. Li F. Hai W.. (2021). Temperature and microbial interactions drive the deterministic assembly processes in sediments of hot springs. Sci. Total Environ. 772:145465. doi: 10.1016/j.scitotenv.2021.145465, PMID: 33571767

[ref23] Hou W. Wang S. Dong H. Jiang H. Briggs B. R. Peacock J. P.. (2013). A comprehensive census of microbial diversity in hot springs of Tengchong, Yunnan Province China using 16S rRNA gene pyrosequencing. PLoS One 8:e53350. doi: 10.1371/journal.pone.0053350, PMID: 23326417 PMC3541193

[ref24] Inskeep W. Jay Z. Tringe S. Herrgard M. Rusch D. (2013). The YNP metagenome project: environmental parameters responsible for microbial distribution in the Yellowstone geothermal ecosystem. Front. Microbiol. 4:67. doi: 10.3389/fmicb.2013.00067, PMID: 23653623 PMC3644721

[ref25] Inskeep W. P. Rusch D. B. Jay Z. J. Herrgard M. J. Kozubal M. A. Richardson T. H.. (2010). Metagenomes from high-temperature chemotrophic systems reveal geochemical controls on microbial community structure and function. PLoS One 5:e9773. doi: 10.1371/journal.pone.0009773, PMID: 20333304 PMC2841643

[ref27] Jungbluth S. P. Bowers R. M. Lin H. T. Cowen J. P. Rappé M. S. (2016). Novel microbial assemblages inhabiting crustal fluids within mid-ocean ridge flank subsurface basalt. ISME J. 10, 2033–2047. doi: 10.1038/ismej.2015.248, PMID: 26872042 PMC5029167

[ref28] Kielak A. M. (2010). Metagenomic analysis of two important, but difficult to culture, soil-borne bacterial phyla: the Acidobacteria and Verrucomicrobia. Amsterdam Vrije Univ. 141, 27–45. doi: 10.4103/0971-5916.154492

[ref29] Kozubal M. Macur R. E. Korf S. Taylor W. P. Ackerman G. G. Nagy A.. (2008). Isolation and distribution of a novel iron-oxidizing crenarchaeon from acidic geothermal springs in Yellowstone National Park. Appl. Environ. Microbiol. 74, 942–949. doi: 10.1128/AEM.01200-07, PMID: 18083851 PMC2258575

[ref30] Lau M. Aitchison J. C. Pointing S. B. (2009). Bacterial community composition in thermophilic microbial mats from five hot springs in Central Tibet. Extremophiles 13, 139–149. doi: 10.1007/s00792-008-0205-3, PMID: 19023516

[ref31] Li T. Weng Y. Ma X. Tian B. Dai S. Jin Y.. (2017). Deinococcus radiodurans toxin-antitoxin MazEF-dr mediates cell death in response to DNA damage stress. Front. Microbiol. 8:1427. doi: 10.3389/fmicb.2017.01427, PMID: 28798741 PMC5526972

[ref32] Li B. Zuza A. V. Chen X. Hu D. G. Shao Z. G. Qi B. S.. (2020). Cenozoic multi-phase deformation in the Qilian Shan and out-of-sequence development of the northernTibetan plateau. Tectonophysics 782-783:228423. doi: 10.1016/j.tecto.2020.228423

[ref33] Lily M. Hu E. Kelsey R. M. Emilie J. S. Madeline T. Alexander J. E.. (2019). Metabolic versatility in a modern lineage of cyanobacteria from terrestrial hot springs. Free Radic. Biol. Med. 140, 224–232. doi: 10.1016/j.freeradbiomed.2019.05.036, PMID: 31163257

[ref34] Liu K. H. Ding X. W. Salam N. Zhang B. Tang X. F. Deng B.. (2018). Unexpected fungal communities in the Rehai thermal springs of Tengchong influenced by abiotic factors. Extremophiles 22, 525–535. doi: 10.1007/s00792-018-1014-y, PMID: 29476252

[ref35] Liu K. H. Ding X. W. Zhang B. Tang X. F. Xiao M. Xian W. D.. (2017). High-throughput sequencing to reveal fungal diversity inhot springs of Rehai at Tengchong in Yunnan. Acta Microbiol. Sin. 57, 1314–1322. doi: 10.13343/j.cnki.wsxb.20170026

[ref36] Lumppio H. L. Shenvi N. V. Summers A. O. Voordouw G. Kurtz D. M. (2001). Rubrerythrin and rubredoxin oxidoreductase in Desulfovibrio vulgar is: a novel oxidative stress protection system. J. Bacteriol. 183, 101–108. doi: 10.1128/JB.183.1.101-108.2001, PMID: 11114906 PMC94855

[ref37] Ma Y. H. Tang B. C. Su S. Y. Zhang S. S. Li C. Y. (2020). Geochemical characteristics of geothermal fluids and water-rock interaction in geothermal reservoirs in and around the Gonghe Basin, Qinghai Province. Earth Sci. Front. 27, 123–133. doi: 10.13745/j.esf.2020.1.14

[ref38] Mao D. Q. Luo Y. Mathieu J. Wang Q. Feng L. Mu Q. H.. (2014). Persistence of extracellular DNA in river sediment facilitates antibiotic resistance gene propagation. Environ. Sci. Technol. 48, 71–78. doi: 10.1021/es404280v, PMID: 24328397

[ref39] Menzel P. Gudbergsdóttir S. R. Rike A. G. Lin L. Zhang Q. Contursi P.. (2015). Comparative metagenomics of eight geographically remote terrestrial Hot Springs. Microb. Ecol. 70, 411–424. doi: 10.1007/s00248-015-0576-9, PMID: 25712554

[ref40] Miller S. R. Strong A. L. Jones K. L. Ungerer M. C. (2009). Bar-coded pyrosequencing reveals shared bacterial community properties along the temperature gradients of two alkaline hot springs in Yellowstone National Park. Appl. Environ. Microbiol. 75, 4565–4572. doi: 10.1128/AEM.02792-08, PMID: 19429553 PMC2704827

[ref41] Mohammadipanah F. Wink J. (2016). Actinobacteria from arid and desert habitats: diversity and biological activity. Front. Microbiol. 6:1541. doi: 10.3389/fmicb.2015.01541, PMID: 26858692 PMC4729944

[ref42] Ng C. C. Chang C. C. Shyu Y. T. (2005). Archaeal community revealed by 16s rRNA and fluorescence in situ hybridization in a sulphuric hydrothermal hot spring, northern Taiwan. World J. Microbiol. Biotechnol. 21, 933–939. doi: 10.1007/s11274-004-6819-4

[ref43] Nishihara A. Matsuura K. Tank M. McGlynn S. E. Thiel V. Haruta S. (2018). Nitrogenase activity in thermophilic chemolithoautotrophic bacteria in the phylum *Aquificae* isolated under nitrogen-fixing conditions from nakabusa hot springs. Microbes Environ. 33, 394–401. doi: 10.1264/jsme2.ME18041, PMID: 30473565 PMC6307999

[ref44] Otaki H. Everroad R. C. Matsuura K. Haruta S. (2012). Production and consumption of hydrogen in hot spring microbial mats dominated by a filamentous anoxygenic photosynthetic bacterium. Microbes Environ. 27, 293–299. doi: 10.1264/jsme2.me11348, PMID: 22446313 PMC4036054

[ref45] Papke R. T. Ramsing N. B. Bateson M. M. Ward D. M. (2003). Geographical isolation in hot spring cyanobacteria. Environ. Microbiol. 5, 650–659. doi: 10.1046/j.1462-2920.2003.00460.x 12871232

[ref46] Peacock J. P. Cole J. K. Murugapiran S. K. Dodsworth J. A. Fisher J. C. Moser D. P.. (2013). Pyrosequencing reveals high-temperature cellulolytic microbial consortia in great boiling spring after in situ lignocellulose enrichment. PLoS One 8:e59927. doi: 10.1371/journal.pone.0059927, PMID: 23555835 PMC3612082

[ref47] Phillips A. A. Speth D. R. Miller L. G. Wang X. T. Wu F. Medeiros P. M.. (2021). Microbial succession and dynamics in meromictic mono Lake, California. Geobiology 19, 376–393. doi: 10.1111/gbi.12437, PMID: 33629529 PMC8359280

[ref48] Podar P. T. Yang Z. M. Bjornsdottir S. H. Podar M. (2020). Comparative analysis of microbial diversity across temperature gradients in hot springs from Yellowstone and Iceland. Front. Microbiol. 11:1625. doi: 10.3389/fmicb.2020.01625, PMID: 32760379 PMC7372906

[ref49] Power J. F. Carere C. R. Lee C. K. Wakerley G. L. J. Evans D. W. Button M.. (2018). Microbial biogeography of 925 geothermal springs in New Zealand. Nat. Commun. 9:2876. doi: 10.1038/s41467-018-05020-y 30038374 PMC6056493

[ref50] Purcell D. Sompong U. Yim L. C. Barraclough T. G. Peerapornpisal Y. Pointing S. B. (2007). The effects of temperature, pH and sulphide on the community structure of hyperthermophilic streamers in hot springs of northern Thailand. FEMS Microbiol. Ecol. 60, 456–466. doi: 10.1111/j.1574-6941.2007.00302.x, PMID: 17386034

[ref51] Rainey F. A. da Costa M. S. (2001). “The genus Thermus” in Bergeys Manual of Systematic Bacteriology. eds. Garrity G. M. Boone D. R. Catenholz R. W., vol. 1 (New York: Springer), 393–404.

[ref52] Ramirez-Arcos S. Fernandez-Herrero L. A. Berenguer J. (1998). A thermophilic nitrate reductase is responsible for the strain specific anaerobic growth of *Thermus thermophilus* HB8. Biochim. Biophys. Acta 1396, 215–227. doi: 10.1016/S0167-4781(97)00183-8, PMID: 9540837

[ref53] Richard L. Robinson M. Palczewska A. Palczewski J. Kidley N. (2017). Comparison of the predictive performance and interpretability of random forest and linear models on benchmark data sets. J. Chem. Inf. Model. 57, 1773–1792. doi: 10.1021/acs.jcim.6b00753, PMID: 28715209

[ref54] Roychoudhury A. N. Cowan D. Porter D. Valverde A. (2013). Dissimilatory sulphate reduction in hypersaline coastal pans: an integrated microbiological and geochemical study. Geobiology 11, 224–233. doi: 10.1111/gbi.12027, PMID: 23374224

[ref04] Schubotz F. Meyer-Dombard D. R. Bradley A. S. Fredricks H. F. Hinrichs K. U. Shock E. L.. (2013). Spatial and temporal variability of biomarkers andmicrobial diversity reveal metabolic and community flexibility in Streamer Biofilm Communities in the Lower Geyser Basin. Yellowstone National Park. Geobiology 11, 549–569. doi: 10.1111/gbi.12051 23981055

[ref55] Schuler C. G. Havig J. R. Hamilton T. L. (2017). Hot spring microbial community composition, morphology, and carbon fixation: implications for interpreting the ancient rock record. Front. Earth Sci. 5, 1–17. doi: 10.3389/feart.2017.00097

[ref56] Sharp C. E. Brady A. L. Sharp G. H. Grasby S. E. Stott M. B. Dunfield P. F. (2014). Humboldt's spa: microbial diversity is controlled by temperature in geothermal environments. ISME J. 8, 1166–1174. doi: 10.1038/ismej.2013.237, PMID: 24430481 PMC4030231

[ref57] Shock E. L. Holland M. Meyer-Dombard D. Amend J. P. Osburn G. R. Fischer T. P. (2010). Quantifying inorganic sources of geochemical energy in hydrothermal ecosystems, Yellowstone National Park, USA. Geochim. Cosmochim. Acta. 74, 4005–4043. doi: 10.1016/j.gca.2009.08.036

[ref58] Song Z. Q. Chen J. Q. Jiang H. C. Zhou E. M. Tang S. K. Zhi X. Y.. (2010). Diversity of Crenarchaeota in terrestrial hot springs in Tengchong, China. Extremophiles 14, 287–296. doi: 10.1007/s00792-010-0307-6, PMID: 20373121

[ref59] Song Z. Q. Wang F. P. Zhi X. Y. Chen J. Q. Zhou E. M. Liang F.. (2013). Bacterial and archaeal diversities in Yunnan and Tibetan hot springs, China. Environ. Microbiol. 15, 1160–1175. doi: 10.1111/1462-2920.12025, PMID: 23126508

[ref60] Tamazawa S. Yamamoto K. Takasaki K. Mitani Y. Hanada S. Kamagata Y.. (2016). *In situ* gene expression responsible for sulfide oxidation and CO2 fixation of an uncultured large sausage-shaped Aquificae bacterium in a sulfidic hot spring. Microbes. Environ. 31, 194–198. doi: 10.1264/jsme2.me16013, PMID: 27297893 PMC4912159

[ref61] Tang X. C. Wang G. L. Ma Y. Zhang D. L. Liu Z. Zhao X.. (2020). Geological model of heat source and accumulation for geothermal anomalies in the Gonghe Basin, northeastern Tibetan plateau. Acta Geol. Sin. 94, 2052–2065. doi: 10.19762/j.cnki.dizhixuebao.2020221

[ref62] Vick T. J. Dodsworth J. A. Costa K. C. Shock E. L. Hedlund B. P. (2010). Microbiology and geochemistry of little Hot Creek, a hot spring environment in the Long Valley caldera. Geobiology 8, 140–154. doi: 10.1111/j.1472-4669.2009.00228.x, PMID: 20002204

[ref63] Vishnivetskaya T. A. Hamilton-Brehm S. D. Podar M. Mosher J. J. Palumbo A. V. Phelps T. J.. (2015). Community analysis of plant biomass-degrading microorganisms from obsidian Pool, Yellowstone National Park. Microbial Ecol. 69, 333–345. doi: 10.1007/s00248-014-0500-8, PMID: 25319238

[ref64] Wang S. Hou W. Dong H. Jiang H. Huang L. Wu G.. (2013). Control of temperature on microbial community structure in hot springs of the Tibetan plateau. PLoS One 8:e62901. doi: 10.1371/journal.pone.0062901, PMID: 23667538 PMC3647046

[ref65] Wang Y. Li P. Guo Q. Jiang Z. Liu M. (2018). Environmental biogeochemistry of high arsenic geothermal fluids. Appl. Geochem. 97, 81–92. doi: 10.1016/j.apgeochem.2018.07.015

[ref66] Wang F. Shi W. Zhang W. Yang L. Wang Y. (2020). Multiple phases of mountain building on the northern Tibetan margin. Lithosphere 2020, 1–16. doi: 10.2113/2020/8829964

[ref67] Wirth R. Sikorski J. Brambilla E. Misra M. Lapidus A. Copeland A.. (2010). Complete genome sequence of thermocrinis albustype strain (HI 11/12T). Stand. Genomic Sci. 2, 194–202. doi: 10.4056/sigs.761490, PMID: 21304702 PMC3035279

[ref05] Yim L. C. Hongmei J. Aitchison J. C. Pointing S. B. (2006). Highly diverse community structure in a remote central Tibetan geothermal spring does not display monotonic variation to thermal stress. FEMS Microbiology Ecology 57, 80–91. doi: 10.1111/j.1574-6941.2006.00104.x 16819952

[ref68] Yuan D. Y. Ge W. P. Chen Z. W. Li C. Y. Wang Z. C. Zhang H. P.. (2013). The growth of northeastern Tibet and its relevance to large-scale continental geodynamics: a review of recent studies. Tectonics 32, 1358–1370. doi: 10.1002/tect.20081

[ref69] Zeytun A. Sikorski J. Nolan M. Lapidus A. Lucas S. Han J.. (2011). Complete genome sequence of *Hydrogenobacter* thermophilustype strain (TK-6T). Stand. Genomic Sci. 4, 131–143. doi: 10.4056/sigs.1463589, PMID: 21677850 PMC3111988

[ref70] Zhang G. W. Guo A. L. Yao A. P. (2004). Western Qinling-Songpan continental tectonic node in China’s continental tectonics. Earth Sci. Front. 11, 23–32.

[ref71] Zhang L. He J. (2012). A novel archaeal phylum: *Thaumarchaeota*-a review. Acta Microbiol Sin. 52, 411–421. doi: 10.13343/j.cnki.wsxb.2012.04.015 22799205

[ref72] Zhang C. Hu S. Zhang S. Li S. Zhang L. Kong Y.. (2019). Radiogenic heat production variations in the Gonghe basin, northeastern Tibetan plateau: implications for the origin of high-temperature geothermal resources. Renew. Energy 148, 284–297. doi: 10.1016/j.renene.2019.11.156

[ref73] Zhang Y. M. Huang L. Q. Jiang H. C. Wu G. (2018a). Hyperthermophilic anaerobic nitrate-dependent Fe(II) oxidization by Tibetan hot spring microbiota and the formation of Fe minerals. Geomicrobiol J. 1-12, 30–41. doi: 10.1080/01490415.2018.1492047

[ref74] Zhang C. Jiang G. Shi Y. Wang Z. Wang Y. Li S.. (2018). Terrestrial heat flow and crustal thermal structure of the gonghe-guide area, northeastern Qinghai-tibetan plateau. Geothermics 72, 182–192. doi: 10.1016/j.geothermics.2017.11.011

[ref75] Zhang H. P. Liu C. C. Xiong J. G. Pang J. Z. Yu J. X. Wang Y. Z. (2022). Late Cenozoic tectonic deformation and geomorphological evolution in the Gonghe-Chaka Basin on the northeastern margin of the Tibetan plateau. Quat. Sci. 42, 662–672. doi: 10.11928/j.issn.1001-7410.2022.03.04

[ref76] Zhang Y. P. Niu Z. G. Zhang Y. Zhang K. (2018). Occurrence of intracellular and extracellular antibiotic resistance genes in coastal areas of Bohai Bay (China) and the factors affecting them. Environ. Pollut. 236, 126–136. doi: 10.1016/j.envpol.2018.01.033, PMID: 29414333

[ref77] Zhang Y. Qi X. Wang S. Wu G. Briggs B. R. Jiang H.. (2020). Carbon fixation by photosynthetic mats along a temperature gradient in a Tengchong hot spring. J. Geophys. Res. Biogeosci. 125, 1–13. doi: 10.1029/2020JG00571936158138

[ref78] Zhang Y. M. Wu G. Jiang H. Yang J. She W. Khan I.. (2018b). Abundant and rare microbial biospheres respond differently to environmental and spatial factors in Tibetan Hot Springs. Front. Microbiol. 9:2096. doi: 10.3389/fmicb.2018.02096, PMID: 30283408 PMC6156277

[ref79] Zhang S. Q. Wu H. D. Zhang Y. Song J. Zhang L. Xu W.. (2020). Characteristics of regional and geothermal geology of the Reshuiquan HDR in Guide County, Qinghai Province. Acta Geol. Sin. 94, 1591–1605. doi: 10.19762/j.cnki.dizhixuebao.2020159

[ref80] Zhao J. Y. Zhen S. J. Zhang C. Y. Yin M. Y. Zhang S. (2021). Composition and functional prediction of microbial communities in deep geothermal water from Jizhong (Central Hebei) geothermal area. Acta Geosci. Sin. 42, 605–616. doi: 10.3975/cagsb.2021.042601

