# Supplementary material for: Bacterial community structure in geothermal springs on the northern edge of Qinghai-Tibet plateau
Source: Front Microbiol. 2023 Jan 31;13:994179. doi: 10.3389/fmicb.2022.994179 (PMC10172933; doi:10.3389/fmicb.2022.994179)
Supplement: Supplementary file 1 [file Data_Sheet_1.PDF]

Supplementary material

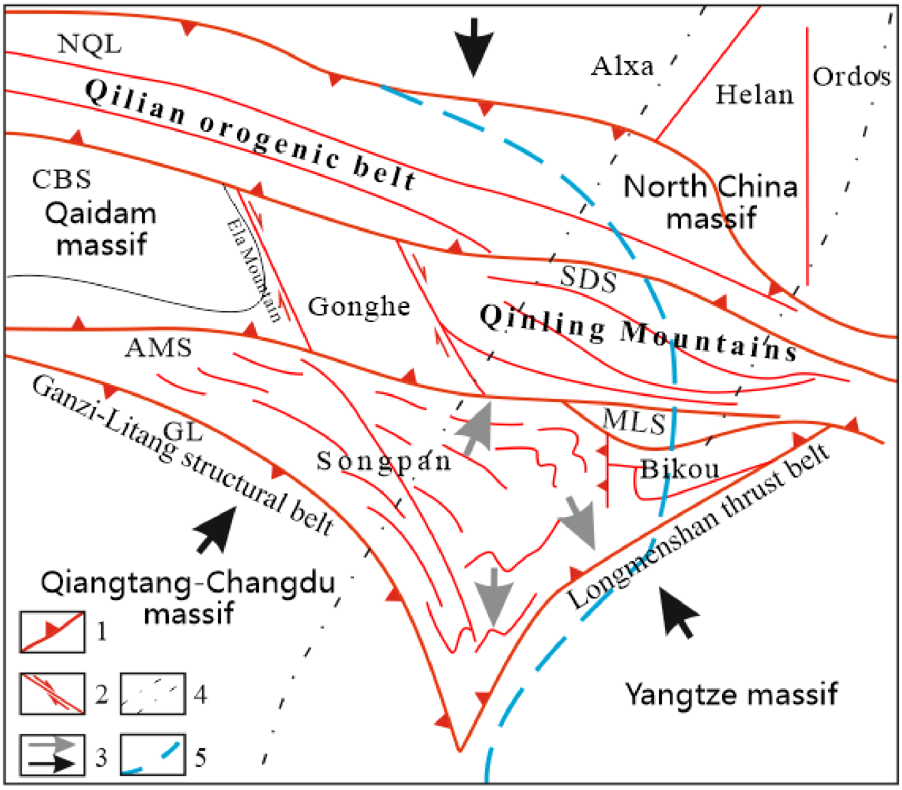

Figure S1 Sketch map of the western Qinling-Songpan tectonic node

(According to Zhang G W et al., 2004; slightly changed)

1 - Main fracture (including paleo-suture belt); 2 - Gonghe Valley tectonic line; 3 - Direction of regional tectonic principal stress; 4 - The East-West boundary of the Helan-Sichuan-Yunnan North-South tectonic belt; 5 - Northeast boundary of Qinghai-Tibet Plateau; CBS-Ancient suture zone in the northern margin of Qaidam Basin; SDS-Shangdan ancient suture zone; KLS-Dongkun Mesozoic suture belt; AMS-Animaqing ancient suture zone; NQL-North Qilian suture zone; MLS-Mianlue ancient suture zone; GL-Ganzi-Litang sewing belt.

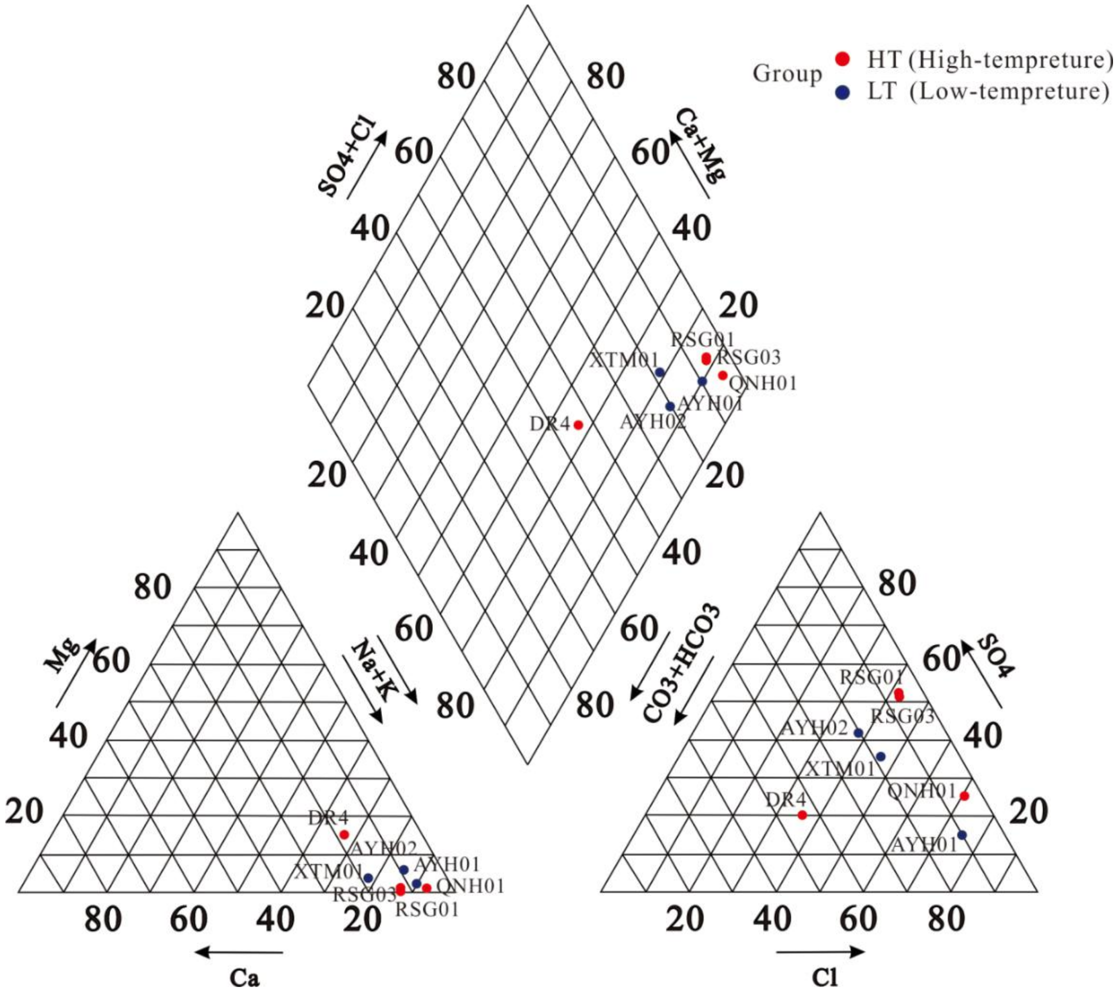

Figure S2 Piper diagram for water type analysis based on the ionic composition

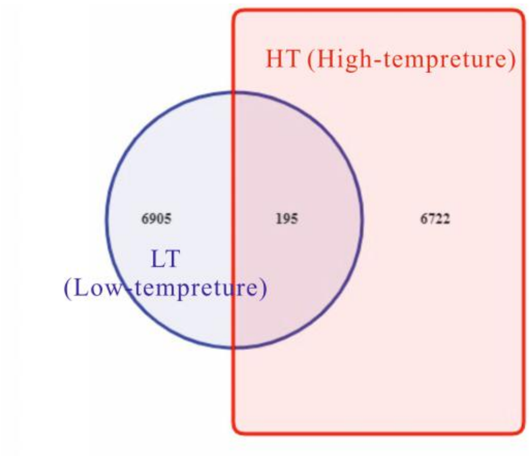

Figure S3 Venn of samples on genus level

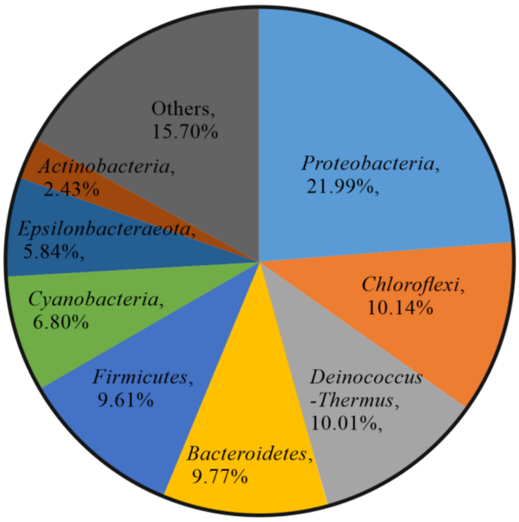

Figure S4 Distribution of common phyla

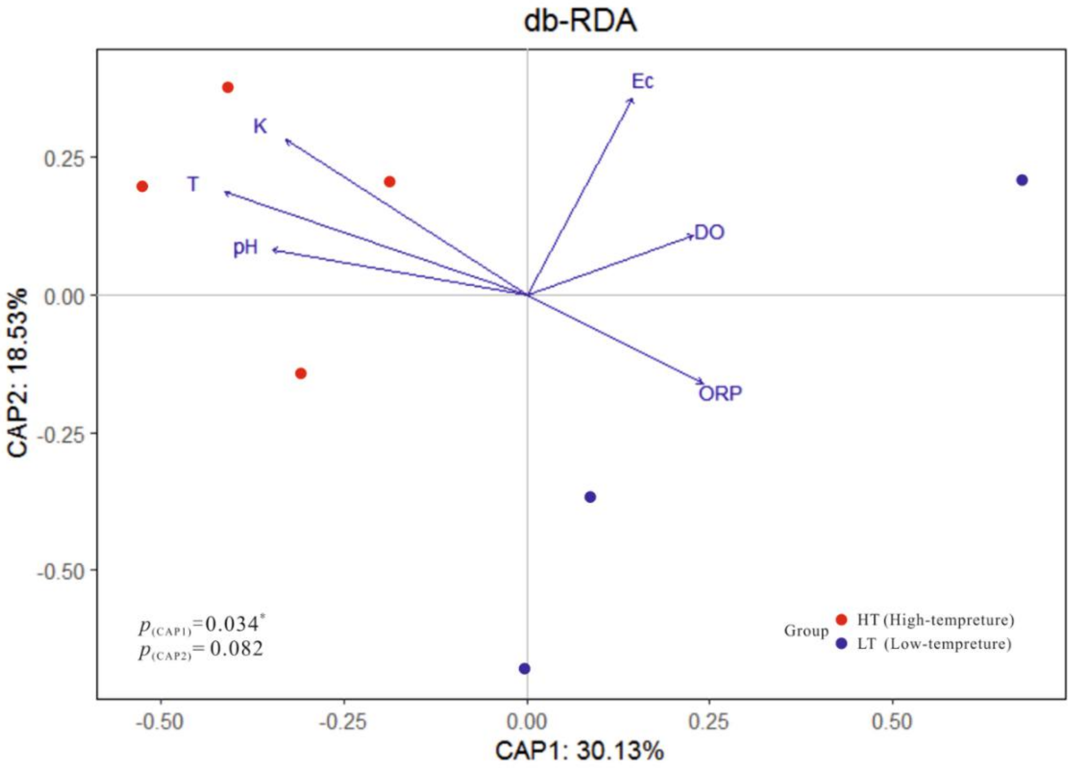

Figure S5 Distance-based Redundancy Analysis (db-RDA) plot showing the relationship of environmental factors to the bacterial community structure

Table S1 The Alpha diversity index of groundwater microbial community from different sampling sites

Group	Sample	Chao1	Shannon	Simpson	Observed_species	Faith_pd	Pielou_e	Goods_coverage
LT (*n*=3)	XTM01	4001.290	9.0021	0.9843	3841.6	223.777	0.7560	0.9890
	AYH01	2582.020	5.8358	0.8536	2328.7	185.822	0.5217	0.9899
	AYH02	1054.350	5.4738	0.8985	991.9	93.203	0.5499	0.9973
	Avg	2545.887	6.7706	0.9121	2387.4	167.601	0.6092	0.9921
HT (*n*=4)	DR401	3813.470	10.5395	0.9971	3670.2	202.453	0.8900	0.9943
	RSG01	3459.800	8.3807	0.9705	3324.3	215.382	0.7164	0.9926
	RSG03	577.360	6.6402	0.9720	567.9	64.450	0.7258	0.9996
	QNH01	273.753	4.7922	0.9164	269	38.486	0.5937	0.9998
	Avg	2031.096	7.5882	0.9640	1957.85	130.193	0.7315	0.9966

Table S2 Correlation analysis between phyla level microorganisms and alpha diversity

	Chao1	Shannon	Simpson	Faith_pd	Pielou_e
Proteobacteria	-0.293	-0.215	-0.317	-0.254	-0.252

Aquificae	-0.402	-0.361	-0.009	-0.427	-0.165
Chloroflexi	0.678	0.848*	0.714	0.565	0.827*
Deinococcus-Thermus	-0.677	-0.374	0.167	-0.698	-0.005
Bacteroidetes	0.43	0.401	0.451	0.38	0.34
Firmicutes	0.316	0.312	0.288	0.266	0.194
Cyanobacteria	-0.131	-0.495	-0.884**	0.008	-0.746
Epsilonbacteraeota	0.097	-0.283	-0.728	0.227	-0.52
Actinobacteria	0.478	0.806*	0.696	0.371	0.868*
Acidobacteria	0.627	0.814*	0.582	0.544	0.764*
Euryarchaeota	0.56	0.478	0.433	0.498	0.368
Planctomycetes	0.904**	0.928**	0.575	0.845*	0.758*
Patescibacteria	0.546	0.287	-0.324	0.611	-0.043
Armatimonadetes	0.015	0.213	0.391	-0.089	0.393
Spirochaetes	-0.265	-0.029	0.31	-0.272	0.153
Thermotogae	-0.575	-0.227	0.234	-0.584	0.108
Verrucomicrobia	0.51	0.675	0.379	0.451	0.594
Synergistetes	0.445	0.364	0.389	0.397	0.284
Dictyoglomi	-0.664	-0.333	0.19	-0.681	0.034
Kiritimatiellaeota	-0.317	-0.357	-0.368	-0.284	-0.431

Note：*:significant correlation, **:extremely significant correlation
